# Improving Clinical Knowledge and Confidence Through a Structured Teaching Programme in Old Age Psychiatry

**DOI:** 10.1192/bjo.2026.11313

**Published:** 2026-06-30

**Authors:** Clara Hollingworth, Salman Siddiqui

**Affiliations:** Pennine Care, Greater Manchester, United Kingdom

## Abstract

**Aims:**

To design, deliver, and evaluate a structured six-month teaching programme for resident doctors and multidisciplinary team (MDT) members within an Old Age Psychiatry service. The objective is to improve clinical knowledge and confidence in key areas of psychiatric care in older adults.

**Methods:**

Teaching topics were selected following consultation with senior clinicians and MDT colleagues to ensure clinical relevance. A monthly teaching programme was then implemented in theOld Agepsychiatry department at Birch Hill Hospital between August 2025 and January 2026, aimed at resident doctors hospital-wide and departmental MDT colleagues. Sessions covered physical health investigations in psychiatry, psychosis in older adults, antipsychotics and metabolic side effects, depression in older adults, clinical cases of rarer dementias, and management of alcohol misuse and insomnia. Structured feedback was collected after each session including Likertscale ratings assessing relevance, usefulness and tutor knowledge, as well as self-rated confidence before and after sessions. Free-text responses were analysed qualitatively using thematic analysis. Numerical data were analysed quantitatively to calculate mean scores, mean differences, and paired t-test statistics in the case of self-rated confidence.

**Results:**

From the 49 feedback survey responses, the programme was highly rated across all domains. Likert scale mean scores (where 1=lowest e.g. not very relevant, 5=highest e.g. very relevant) showed 4.90 for relevance, 4.83 for usefulness, and 4.86 for tutor understanding. Self-rated confidence increased from a mean of 3.39 pre-teaching session to a mean of 4.55 post-teaching session, where there was a statistically significant difference (p<0.001) with a large effect size (*d*=1.07). Qualitatively, thematic analysis identified overarching themes including high clinical relevance, improved understanding of clinical presentations, greater knowledge of pharmacology and medications, appreciation of clear and structured teaching, and the value of group engagement for team cohesiveness.

**Conclusion:**

This six-month structured teaching programme improved knowledge and confidence on a variety of clinical topics among resident doctors and MDT colleagues inOldAgepsychiatry. Qualitative themes highlighted that the programme was very well received and also showed the value of group discussion in teaching for team building, demonstrating broader benefits of such educational interventions. This teaching programme model should be replicated across other services and extended within this department to encourage the development of highly knowledgeable and confident clinicians.

